# Long‐term clinical, virological and immunological outcomes following planned treatment interruption in HIV‐infected children

**DOI:** 10.1111/hiv.12986

**Published:** 2020-10-29

**Authors:** R Freguja, A Bamford, M Zanchetta, P Del Bianco, C Giaquinto, L Harper, A Dalzini, TR Cressey, A Compagnucci, Y Saidi, Y Riault, D Ford, D Gibb, N Klein, A De Rossi, the Paediatric European Network for Treatment of AIDS (PENTA) 11 Trial Team

**Affiliations:** ^1^ Section of Oncology and Immunology Department of Surgery, Oncology and Gastroenterology University of Padova Padova Italy; ^2^ Department of Paediatric Infectious Diseases Great Ormond Street Hospital for Children NHS Trust London UK; ^3^ UCL Great Ormond Street Institute of Child Health London UK; ^4^ MRC Clinical Trials Unit London UK; ^5^ Immunology and Molecular Oncology Unit Veneto Institute of Oncology IOV – IRCCS Padova Italy; ^6^ Clinical Trials and Biostatistic Unit Veneto Institute of Oncology IOV – IRCCS Padova Italy; ^7^ Department of Mother and Child Health University of Padova Padova Italy; ^8^ PHPT/IRD 174 Faculty of Associated Medical Sciences Chiang Mai University Chiang Mai Thailand; ^9^ Department of Immunology & Infectious Diseases Harvard T.H Chan School of Public Health Boston MA USA; ^10^ Department of Molecular & Clinical Pharmacology University of Liverpool Liverpool UK; ^11^ INSERMSC10‐US019 Essais thérapeutiques et maladies Infectieuses Villejuif France

**Keywords:** ARV, children, HIV, immunology, treatment interruption, virology

## Abstract

**Objectives:**

Planned treatment interruption (PTI) of antiretroviral therapy (ART) in adults is associated with adverse outcomes. The PENTA 11 trial randomized HIV‐infected children to continuous ART (CT) *vs*. CD4‐driven PTIs. We report 5 years’ follow‐up after the end of main trial.

**Methods:**

Post‐trial, all children resumed ART. Clinical, immunological, virological and treatment data were collected annually. A sub‐study investigated more detailed immunophenotype. CT and PTI arms were compared using intention‐to‐treat. Laboratory parameters were compared using linear regression, adjusting for baseline values; mixed models were used to include all data over time.

**Results:**

In all, 101 children (51 CT, 50 PTI) contributed a median of 7.6 years, including 5.1 years of post‐trial follow‐up. Post‐trial, there were no deaths, one pulmonary tuberculosis and no other CDC stage B/C events. At 5 years post‐trial, 90% of children in the CT *vs*. 82% in the PTI arm had HIV RNA < 50 copies/mL (*P* = 0.26). A persistent increase in CD8 cells was observed in the PTI arm. The sub‐study (54 children) suggested that both naïve and memory populations contributed to higher CD8 cells following PTI. Mean CD4/CD8 ratios at 5 years post‐trial were 1.22 and 1.08 in CT and PTI arms, respectively [difference (CT – PTI) = −0.15; 95% CI: −0.34–0.05), *P* = 0.14]. The sub‐study also suggested that during the trial and at early timepoints after the end of the trial, reduction in CD4 in the PTI arm was mainly from loss of CD4 memory cells.

**Conclusions:**

Children tolerated PTI with few long‐term clinical, virological or immunological consequences.

## Introduction

Studies of planned treatment interruption (PTI) of antiretroviral therapy (ART) have aimed to reduce ART exposure, toxicity and development of resistance [1, 2, 3]. Results in adult studies demonstrated poor immunological and virological outcomes and higher rates of opportunistic infections, non‐AIDS events and mortality [4, 5, 6, 7]. The Paediatric European Network for Treatment of AIDS (PENTA) 11 trial was a randomized phase II trial investigating clinical and immunological responses to PTIs in HIV‐1‐infected children aged 2–15 years, 26% CDC category C disease [8]. Children were randomized to continuous therapy (CT) or PTI until a predefined CD4 cut‐off was reached, a CDC B/C clinical event occurred or 48 weeks of PTI were completed. In the PTI arm, after 24 weeks back on continuous ART, children whose CD4 had recovered were eligible for a second PTI.

Results from the main trial showed that in the PTI arm, there were no significant increases in clinical adverse outcomes. HIV‐1 plasma viraemia increased rapidly. After an initial decline following ART interruption, CD4 cell counts remained stable up to 48 weeks off ART in most children, and naïve and memory cell proportions remained constant [8, 9]. This contrasts with adults in whom an initial rapid CD4 decrease was followed by a steady lower rate of decline [3]. In PENTA 11, younger age and higher nadir CD4 were associated with better recovery following PTI [8]. PTI was associated with increases in CD4 cells expressing Ki67 and human leucocyte antigen‐DR and increased cell‐associated HIV‐1 DNA [9].

After the main trial, children were restarted ART and were followed up long‐term. We have previously reported outcomes at 2 years after the end of main trial [10]. Here we describe 5‐year outcomes.

## Methods

Our methods were as previously described [8, 9, 10]. HIV‐infected children were eligible if they had been on three‐drug ART for at least 24 weeks, had HIV‐1 RNA < 50 copies/mL and two recent CD4% of at least 30% (age 2–6 years), or, alternatively, a CD4% of at least 25% and a CD4 cell count of at least 500 cells/μL (age 7–15 years). Randomization was stratified by age of ART initiation, age and lowest recorded pre‐ART CD4. Patients were followed until the last randomized child had completed 72 weeks’ follow‐up.

The main trial ended in May 2008 and ART re‐initiation was recommended in the PTI arm. Routine annual data (ART, AIDS events, important clinical events, weight/height, CD4 and CD8 count/percentage, HIV‐1 RNA viral load) were collected for 5 years from end of the main trial. The last data from long‐term follow‐up were collected in April 2014. Full ethics committee approval was obtained at each participating centre (summarized in previous reports [8, 9, 10]). Informed consent was obtained at the main study entry and additional consent was obtained at the beginning of long‐term follow‐up.

In centres able to separate and store cells, 10 mL whole blood was collected in EDTA annually. Peripheral blood mononuclear cells (PBMCs) were isolated by density gradient centrifugation and frozen for storage. Children with at least two PBMC samples available during 5 years’ follow‐up were included in the sub‐study aimed at providing in‐depth cell phenotyping and virological data for exploratory analysis.

### Sub‐study laboratory methods

#### HIV‐1 DNA quantification

Cell‐associated HIV‐1 DNA was measured in PBMC by real‐time polymerase chain reaction (PCR), as previously described [11]. HIV‐1 copy number was normalized against the housekeeping gene telomerase reverse transcriptase (TERT) and results were expressed as HIV‐1 DNA copies/10^6^ PBMCs [12].

#### Intracellular HIV‐1 RNA quantification

RNA was extracted from 3 × 10^6^ PBMCs using Trizol Reagent (Invitrogen, Carlsbas, CA, USA). Trizol (500 µL) and quantitation standard (7 µL; internal control; Roche Diagnostic Systems, Branchburg, NJ, USA) were added to PBMCs. Samples were incubated with 200 µL chloroform for 15 min on ice. After centrifugation, RNA was stored at −20°C overnight with cold isopranolol. Samples were centrifuged and supernatant removed. Each RNA pellet was resuspended in 75 µL of elution buffer heated to 70°C. HIV‐1 RNA levels were determined by real‐time PCR with the Amplicor HIV‐1 Monitor Test using Cobas TaqMan48 (Roche Diagnostic Systems) [13, 14].

#### Immunophenotyping

Aliquots of frozen samples were thawed and cell suspensions incubated for 15 min in the dark with labelled monoclonal antibodies (Beckton Dickinson Bioscience Pharmingen, San Diego, CA, USA): anti‐CD3 fluoroscein isothiocyanate, anti‐CD4 peridinin chlorophyll protein (PerCP), anti‐CD8 PerCP, anti‐CD38 phycoerythrin (PE), anti‐CD45RO allophyocyanin (APC) and anti‐CD45RA APC. Appropriate isotype controls (mouse IgG1‐PE and mouse IgG2b‐APC) were used to evaluate non‐specific staining. Cells were washed with Automacs Buffer (Miltenyi Biotec Inc., Auburn, CA, USA) and resuspended in phosphate‐buffered saline supplemented with 1% paraformaldehyde. Samples were analysed by four‐colour flow cytometry using a Calibur fluorescence‐activated cell sorter (Beckton Dickinson) equipped with a 488‐nm argon‐ion laser and a 635‐nm red diode laser. In all, 50 000 events were collected in the lymphocyte gate according to forward and side scatter [15]. Data were processed using CellQuest Pro Software (Becton Dickinson) and analysed using Kaluza software v.1.2 (Beckman Coulter).

#### TREC quantification

Thymic output in PBMCs was assessed using T‐cell receptor excision circle (TREC) levels measured by real‐time PCR [11, 16], expressed as TREC copies/10^5^ PBMCs [16, 17].

### Statistical methods

#### Main study

Laboratory measurements at end of main trial were defined as those nearest to but no more than 3 months prior to 29 May 2008 and annually (up to end of the fifth year) thereafter as those taken nearest to but within ±3 months of these respective time points.

Comparison between the two randomized arms was according to intention‐to‐treat, adjusting for stratification factors in regression analyses. Rates of clinical events from end of main trial to last clinic visit were compared using a random‐effects Poisson model allowing more than one event per child. Virological suppression < 50 copies/mL and CD4/CD8 ratio < 1 were analysed using Poisson regression with robust error variance. CD4 and CD8 percentages and counts and CD4/CD8 ratio were analysed using linear regression, adjusting for values at randomization. Mixed‐effects models were fitted over time, including all measurements in the CT arm and measurements after restarting treatment after the last PTI in the PTI arm. Cubic splines were used to investigate the shape of the curves over time by arm; curves were then approximated by a linear model in the CT arm and a piecewise linear model in the PTI arm, allowing for a different slope for the first 3 months after restarting continuous treatment. For continuous outcomes, heteroscedastic random effects were modelled by arm with random effects for intercept and slope in the CT arm (unstructured covariance matrix), random effects for intercept and slope in the PTI arm (unstructured covariance matrix) and an additional random effect for the slope in the first 3 months after restarting treatment in the PTI arm. For virological suppression < 50 copies/mL, two random effects were included, one for each arm; there was too little variability by slope to include additional random effects. Time 0 was defined as the end of the main trial (prior measurements were given negative time). Models were extrapolated as necessary to estimate where curves would cross (after those in the PTI arm had been back on continuous treatment for at least 3 months), indicating that laboratory values in the PTI arm had reached those in the CT arm. Two‐sided 95%‐based confidence intervals of the difference between arms at different time points from the end of main trial and for the time point where the PTI and CT curves would cross were obtained using 1000 bootstrap samples.

Based on all measurements of CD4/CD8 ratio in the PTI arm after each child restarted ART following the most recent PTI, effects of the following factors on CD4/CD8 ratio recovery were assessed: baseline characteristics, CD4/CD8 ratio at ART re‐initiation, RNA plasma viraemia at ART re‐initiation, nadir CD4% and number of PTIs. Mixed‐effects models were fitted as described earlier, although time 0 was defined as the time of restarting continuous ART; effects of predictors were similar when time 0 was defined as the end of the main trial, as previously (data not shown). All models were adjusted for time since restarting continuous ART and baseline (trial entry) CD4/CD8 ratio. The final multivariable model included all factors significant at *P* < 0.05 in univariable analyses. Current RNA was then added to the final model as a surrogate for recent adherence to ART. Data were analysed using STATA v.13.

#### Sub‐study

This was an exploratory analysis using samples available from children followed up in centres able to store PBMCs. Results were included if a child had at least two samples available in the 5‐year period following the end of the main trial. Samples were assigned to their nearest annual time point to maximize the number of samples included.

Mixed‐effects linear models were used to examine change over time in binary and continuous outcomes within and between CT and PTI groups, using mixed‐effects Poisson and linear regression models, respectively, as for the main trial. The models included as explanatory variables the number of years since the end of the main trial, as a categorical variable, the treatment group and their interaction, in addition to a compound symmetry covariance structure for the random effects for intercept and slope. Data were analysed using SAS v.9.2 and STATA v.13.0.

## Results

### Main study

A total of 101 (51 CT, 50 PTI) out of 109 children in the main trial participated in long‐term follow‐up, including 79 from Europe and 22 from Thailand [10]. Characteristics at trial enrolment were similar by arm (Table S1).

Five‐year post‐trial follow‐up forms were completed for 47 (92%) of the children on CT and 46 (92%) of the children on PTI. Median (IQR) duration of follow‐up from trial enrolment was 7.6 (6.9–8.2), a median of 5.1 (4.9–5.3) years after main trial end. Fifteen out of 50 PTI children had experienced two PTIs during the main trial. At the end of the main trial, one CT child was off ART, 43 on PTI re‐initiated ART for a median (IQR) duration of 11.8 (7.1–22.8) months, six were undergoing a PTI and one child was off ART.

#### Clinical outcomes during 5 years of follow‐up after the main trial

There were no deaths. One PTI child in Thailand had pulmonary TB (this was diagnosed 4.4 years after the end of the trial); there were no other new CDC stage B or C events. There were 33 clinical events diagnosed in 18 (35%) CT children and 32 events in 13 (26%) PTI children. The rates [95% confidence interval (CI)] of events per 100 child‐years were 13.1 (9.3–18.5) in the CT arm and 13.0 (9.2–18.4) in the PTI arm (rate ratio comparing PTI *vs*. CT of 0.91; 95% CI: 0.40–2.09; *P* = 0.83), with similar events in the two arms. Events included infections/infestations (*n* = 13 total), gastrointestinal disorders (*n* = 9), respiratory illness (*n* = 6), blood lipid abnormalities (*n* = 6), anaemia/thrombocytopenia (*n* = 5) and others (*n* = 26).

#### Laboratory outcomes during 5 years of follow‐up after the main trial

Immunology and virology results from the main study are summarized in Fig. S1. At the end of the main trial, PTI children were less likely to have undetectable plasma viraemia (HIV‐1 RNA < 50 copies/mL) than CT children (65% *vs*. 87%; *P* = 0.02; Table 1); by 2 years the proportions were similar (84% *vs*. 88%; *P* = 0.57). Based on a mixed model including all measures, proportions suppressed were predicted to be the same in the two arms by 4.9 years (95% CI: −0.7–∞) from the end of the main trial.

**Table 1:** Summary of virological and immunological outcomes at 5 years after the end of the main trial

	CT			PTI			PTI *vs*. CT	*P* (adj^†^)
	*N*	Mean or proportion	SE	*N*	Mean or proportion	SE	Difference or IRR (adj^†^)	
HIV RNA < 50 copies/mL								
EOT	45	87%	5%	46	65%	7%	0.74 (0.59–0.94)	0.02
1 year	49	90%	4%	47	77%	6%	0.85 (0.71–1.02)	0.07
2 years	48	88%	5%	49	84%	5%	0.95 (0.81–1.12)	0.57
3 years	46	87%	5%	47	87%	5%	1.01 (0.87–1.18)	0.89
4 years	44	89%	5%	43	86%	5%	0.97 (0.83–1.14)	0.72
5 years	41	90%	5%	38	82%	6%	0.90 (0.74–1.09)	0.26
CD4%								
EOT	46	35.8	0.9	46	31.4	0.9	−4.9 (−7.5 to −2.4)	<.001
1 year	46	35.7	1.0	50	32.7	1.0	−3.5 (−6.2 to −0.7)	0.01
2 years	48	36.2	1.0	49	34.5	1.0	−1.9 (−4.7–0.9)	0.19
3 years	46	36.9	1.2	45	35.8	1.2	−1.3 (−4.7–2.1)	0.45
4 years	39	37.4	1.2	43	35.8	1.2	−2.1 (−5.5–1.2)	0.21
5 years	39	36.6	1.1	36	35.6	1.1	−1.0 (−4.2–2.2)	0.53
CD4								
EOT	46	931	36	40	849	39	−102 (−208–4)	0.06
1 year	45	926	45	47	805	44	−129 (−255 to −4)	0.04
2 years	49	877	38	48	844	39	−40 (−146–66)	0.45
3 years	46	850	41	47	789	41	−65 (−182–52)	0.27
4 years	41	819	46	42	838	46	2 (−126–131)	0.97
5 years	40	809	38	34	780	40	−36 (−145–73)	0.51
CD8%								
EOT	44	34.2	1.4	44	37.6	1.4	3.8 (−0.2–7.9)	0.06
1 year	45	33.6	1.4	48	36.7	1.3	3.5 (−0.4–7.3)	0.08
2 years	47	33.7	1.4	49	36.3	1.4	2.8 (−1.2–6.8)	0.17
3 years	44	33.8	1.5	44	34.8	1.5	0.7 (−3.4–4.8	0.74
4 years	38	32.3	1.4	42	36.0	1.3	4.0 (0.0–7.9)	0.05
5 years	38	33.4	1.6	35	36.7	1.6	3.2 (−1.5–7.9)	0.18
CD8								
EOT	44	907	72	39	1074	76	191 (−24–405)	0.08
1 year	44	869	52	45	914	52	53 (−95–202)	0.48
2 years	47	824	58	46	922	59	103 (−64–269)	0.22
3 years	44	721	39	43	822	39	99 (−11–210)	0.08
4 years	38	693	49	39	850	49	156 (14–299)	0.03
5 years	38	755	53	32	820	57	55 (−106–216)	0.50
CD4/CD8								
EOT	44	1.19	0.06	44	0.90	0.06	−0.31 (−0.48 to −0.14)	<.001
1 year	45	1.19	0.05	48	0.99	0.05	−0.22 (−0.37 to −0.08)	0.003
2 years	47	1.18	0.06	49	1.09	0.06	−0.10 (−0.27–0.06)	0.22
3 years	44	1.25	0.06	46	1.13	0.06	−0.12 (−0.30–0.05)	0.17
4 years	38	1.28	0.07	43	1.11	0.06	−0.19 (−0.37 to −0.01)	0.04
5 years	38	1.22	0.07	35	1.08	0.07	−0.15 (−0.34–0.05)	0.14
CD4/CD8 < 1								
EOT	44	34%	7%	44	64%	7%	1.98 (1.26–3.11)	0.003
1 year	45	31%	7%	48	58%	7%	1.99 (1.24–3.20)	0.005
2 years	47	34%	7%	49	55%	7%	1.70 (1.08–2.66)	0.02
3 years	44	23%	6%	46	43%	7%	1.99 (1.08–3.70)	0.03
4 years	38	24%	7%	43	37%	7%	1.67 (0.83–3.34)	0.15
5 years	38	29%	7%	35	46%	8%	1.70 (0.93–3.12)	0.08

CT, continuous antiretroviral therapy (ART); PTI, planned treatment interruption of ART; IRR, incidence rate ratio; EOT, end of trial.

^†^ Adjusted for stratification factors and value at trial entry.

Mean CD4% was lower in PTI children at end of main trial [adjusted difference (PTI – CT) = −4.9%, 95% CI: −7.5 to −2.4; *P* < 0.001). By 2 years, the difference between arms was borderline (−1.8%, 95% CI: −4.6–1.0; *P* = 0.20), but the mixed model suggested the difference persisted beyond 2 years with mean values in the two arms predicted to be the same at 7.2 years (95% CI: 2.7–34.9). Results were similar for absolute CD4 count (Table 2) with the mean values in the two arms predicted to be the same at 5.0 years (95% CI: 2.2–14.0). Mean CD8% and CD8 were higher in PTI children at the end of the main trial and there was some suggestion that differences persisted longer for CD8% and absolute CD8 count (Tables 1, 2); although mean values in the two arms were predicted to be same at 11.0 and 18.3 years respectively, CIs did not preclude CD8% and CD8 count in the PTI arm not returning to the levels in the CT arm.

**Table 2:** Modelling the effect of planned treatment interruptions on long‐term CD4 and CD8 measures

	Difference between arms (PTI – CT)^†^ (95% CI)	Time after EOT (years) at which means in the PTI and CT arms are predicted to be the same^†^ (95% CI)
CD4%		
EOT	−4 (−5 to −2)	
2 years	−3 (−5 to −1)	
5 years	−1 (−4–2)	
Curves cross (years)		7.2 (2.7–34.9)
CD4		
EOT	−115 (−179 to −54)	
2 years	−69 (−136 to −6)	
5 years	1 (−92–87)	
Curves cross (years)		5.0 (2.2–14.0)
CD8%		
EOT	4.1 (1.7–6.3)	
2 years	3.3 (0.6–6.2)	
5 years	2.2 (−1.2–6.1)	
Curves cross (years)		11.0 (3.1–∞^‡^)
CD8		
EOT	106 (0–205)	
2 years	95 (1–186)	
5 years	77 (−26–170)	
Curves cross (years)		18.3 (1.7–∞^§^)
CD4/CD8		
EOT	−0.27 (−0.36 to −0.15)	
2 years	−0.19 (−0.30 to −0.07)	
5 years	−0.09 (−0.24–0.07)	
Curves cross (years)		7.4 (3.6–18.6)

CT, continuous antiretroviral therapy (ART); PTI, planned treatment interruption of ART; IRR, incidence rate ratio; EOT, end of trial.

^†^ Adjusted for stratification factors and value at trial entry.

^‡^ Curves did not cross in 6% of bootstrap samples.

^§^ Curves did not cross in 24% of bootstrap samples.

The CD4/CD8 ratio is an additional important marker of immune competence [18, 19, 20]. The mean CD4/CD8 ratio was lower at the end of the main trial: 64% of PTI children had CD4/CD8 ratio < 1 compared with 34% of the CT children (*P* = 0.003); by 5 years the corresponding proportions were 46% *vs*. 29% (*P* = 0.08). Mean CD4/CD8 ratios at 5 years post‐trial were 1.22 and 1.08 in CT and PTI arms, respectively [difference (CT – PTI) = −0.15; 95% CI: −0.34–0.05; *P* = 0.14) (Table 1). In both arms the CD4/CD8 ratio at 5 years was strongly associated with the CD4/CD8 ratio at the end of the main trial: in the PTI arm, 12/20 participants with CD4/CD8 ratio < 1 at the end of the trial remained < 1 at 5 years, and only 3/14 participants with CD4/CD8 ratio ≥ 1 at the end of the trial were < 1 at 5 years (Fisher’s exact for association, *P* = 0.03); corresponding proportions in the CT arm were 6/10 and 4/23 (*P* = 0.01). The mixed model predicted CD4/CD8 ratio to be the same in the two arms at 7.4 years (95% CI: 3.6–18.6) (Table 2).

#### Predictors of earlier CD4/CD8 recovery after restarting antiretroviral therapy

Following re‐initiation of ART after the last PTI, CD4/CD8 recovery was strongly associated with duration of ART since re‐initiation and baseline CD4/CD8 ratio (Table 3). After adjustment for these predictors, the only factor that remained significantly associated with higher CD4/CD8 in the multivariable model was the minimum CD4/CD8 ratio before ART re‐initiation. Although most recent HIV‐1 RNA plasma viraemia (fitted as a surrogate for treatment adherence) was associated with CD4/CD8 recovery in the multivariable model, including it had a minimal effect on the impact of other predictors.

**Table 3:** Associations with recovery of CD4/CD8 ratio after restarting antiretroviral therapy (ART) following the most recent planned treatment interruption of ART(*n* = 49)

	Univariable analyses^§^		Multivariable analysis I^¶^		Multivariable analysis II^#^	
	Difference in mean absolute CD4/CD8 ratio (95% CI)	*P*	Difference in mean absolute CD4/CD8 ratio (95% CI)	*P*	Difference in mean absolute CD4/CD8 ratio (95% CI)	*P*
Baseline CD4/CD8 ratio (per unit)^‡^	0.35 (0.17–0.52)	< 0.001	0.24 (0.11–0.37)	< 0.001	0.25 (0.13–0.38)	< 0.001
Time since ART re‐initiation^‡^ (months; initial 3 months^†^)	0.13 (0.10–0.16)	< 0.001	0.12 (0.09–0.16)	< 0.001	0.10 (0.07–0.13)	< 0.001
Time since ART re‐initiation^‡^ (months)	0.002 (0.001–0.004)	< 0.001	0.002 (0.001–0.004)	< 0.001	0.002 (0.001–0.004)	< 0.001
Age at baseline (per year)	−0.01 (−0.03–0.01)	0.50				
Sex						
Male	0					
Female	−0.10 (−0.25–0.04)	0.15				
Ethnicity						
White	0					
Black	−0.01 (−0.19–0.17)					
Asian	0.02 (−0.18–0.22)					
Other	−0.10 (−0.33–0.14)	0.84				
Disease stage at baseline						
A/N	0					
B	−0.07 (−0.23–0.10)					
C	−0.02 (−0.21–0.18)	0.73				
Age child started ART						
< 1 year	0		0		0	
1–5 years	−0.20 (−0.36 to −0.04)		−0.12 (−0.24–0.01)		−0.11 (−0.23–0.01)	
≥ 5 years	−0.05 (−0.23–0.13)	0.035	−0.01 (−0.16–0.13)	0.10	−0.01 (−0.15–0.13)	0.14
Cumulative ART exposure up to baseline (months)	−0.01 (−0.03–0.02)	0.67				
Baseline weight‐for‐age *z* score (per unit)	−0.02 (−0.08–0.04)	0.51				
Nadir CD4% (per 10%)	0.06 (0.00–0.12)	0.046	0.02 (−0.03–0.06)	0.49	0.02 (−0.02–0.07)	0.37
Number of planned treatment interruptions child underwent						
1	0					
2	0.08 (−0.09–0.25)	0.38				
Lowest CD4/CD8 ratio before ART re‐initiation (per unit)	0.82 (0.61–1.03)	< 0.001	0.78 (0.57–0.99)	< 0.001	0.76 (0.55–0.96)	< 0.001
CD4/CD8 ratio at ART re‐initiation (per unit)	0.72 (0.52–0.92)	< 0.001				
HIV RNA at ART re‐initiation (per log_10_ copies/mL)	0.01 (−0.11–0.12)	0.90				
Most recent HIV RNA (per log_10_ copies/mL)	‐	‐			–0.08 (−0.10 to −0.06)	< 0.001

^†^ Additional effect in the first 3 months (value = 3 after ≥ 3 months have elapsed after re‐initiation).

^‡^ Adjusted for in all models.

^§^ Baseline CD4/CD8 ratio and covariates for time since ART re‐initiation were fitted together; other factors are adjusted for these covariates.

^¶^ Multivariable analysis I includes baseline CD4/CD8 ratio, covariates for time since ART re‐initiation and other factors significant in the univariable analysis (*P* < 0.05); lowest CD4/CD8 ratio before ART re‐initiation and CD4/CD8 ratio at ART re‐initiation were highly correlated (*r* = 0.92) and only the former was included.

^#^ Factors as in multivariable analysis I with time‐updated HIV RNA (surrogate for recent adherence to ART).

Models fitted for CD4% and CD8% recovery resulted in similar conclusions; in the corresponding multivariable model significant predictors (*P* < 0.05) of CD4% (CD8%) included duration of ART since re‐initiation, baseline CD4% (CD8%) and lowest CD4% (highest CD8%) before ART re‐initiation (data not shown).

### Sub‐study

Fifty‐four (23 CT, 31 PTI) children were included in the sub‐study. There were few differences at trial baseline between the children in the sub‐study and the remainder: ethnicity differed primarily because Thai children did not participate; baseline weight‐for‐age and years on ART were marginally higher in sub‐study participants (Table S2). Of 31 PTI children, 20 underwent one interruption, and 11 underwent two interruptions. Median (IQR) follow‐up from trial enrolment in sub‐study participants was 7.4 (3.7–8.5) years.

**Fig. 1:**
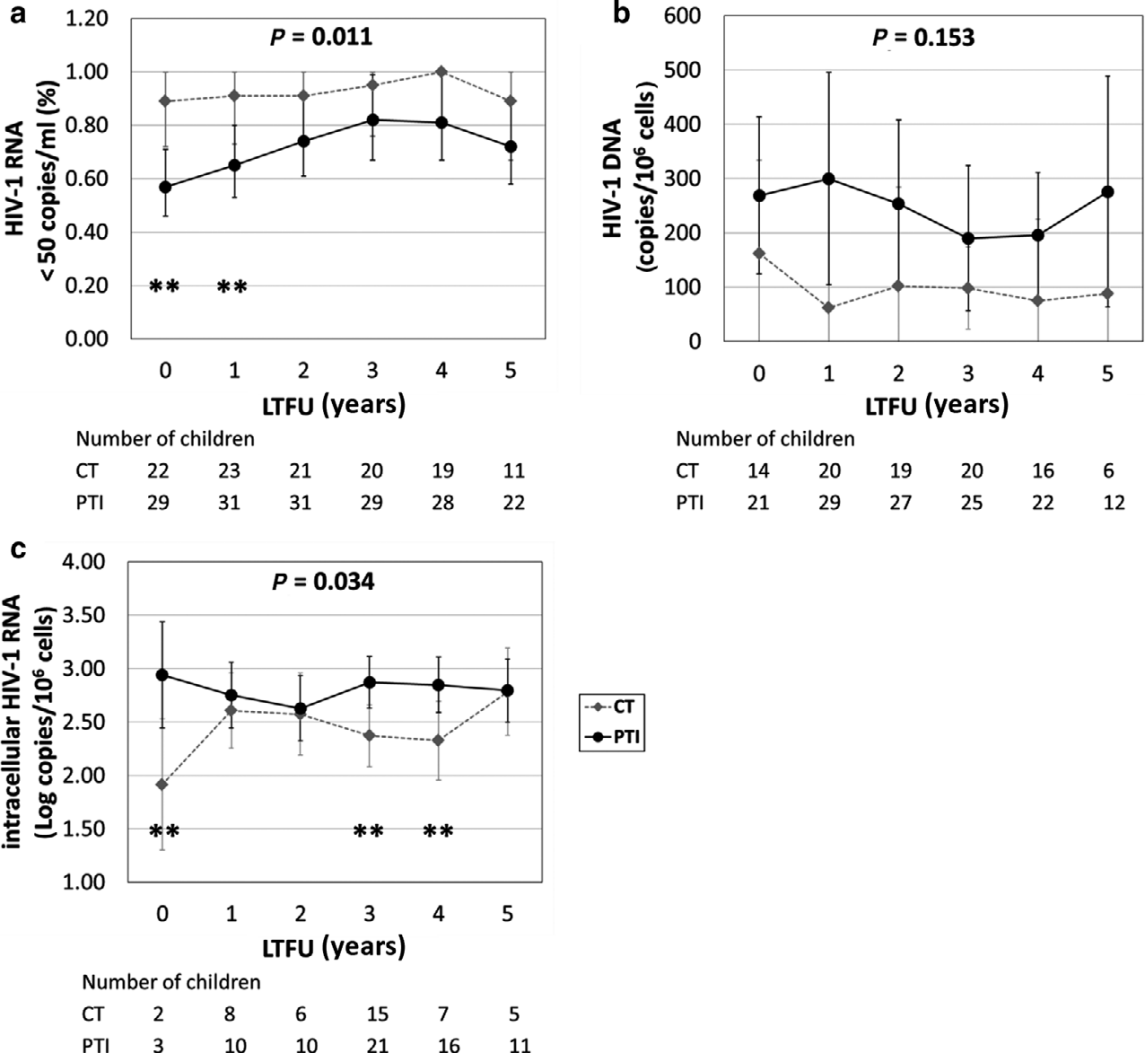
Trends in HIV‐1 plasma viraemia, HIV‐1 DNA and intracellular HIV‐RNA during 5 years of long‐term follow‐up (LTFU) in children on continuous antiretroviral (ART) therapy (CT) and those undergoing planned treatment interruption (PTI) of ART. (a) Proportion of children with undetectable HIV‐1 plasma viraemia; (b) levels of HIV‐1 DNA; (c) levels of intracellular HIV‐1 RNA. **P < 0.05; *P < 0.10; *P*‐values in panel: overall difference between PTI and CT groups. Whiskers represent 95% confidence interval of mean values.

Over 5 years of post‐trial follow‐up, the difference in HIV‐1 plasma viraemia between PTI and CT was significant (*P* = 0.011). Sub‐study PTI children were less likely to be suppressed at the end of the main trial (57%, of PTI *vs*. 90% of CT had undetectable plasma viraemia; *P* = 0.005). This persisted at 1 year (65% PTI *vs*. 90% CT; *P* = 0.027), but differences thereafter were non‐significant (Fig. 1a). HIV‐1 DNA levels were higher in the PTI arm at end of main trial [mean (95% CI) of 269 (124–414) copies/10^6^ PBMCs in PTI *vs*. 162 (0–334) copies/10^6^ PBMCs in CT]; levels thereafter remained higher, although at non‐significant level, in PTI children (Fig. 1b). Intracellular HIV‐1 RNA levels were higher in PTI than in CT children at the end of the main trial (*P* = 0.012), and thereafter (at 3 and 4 years), but non‐significantly different at 5 years (Fig. 1c).

#### CD4 and CD8 cell phenotypes

**Fig. 2:**
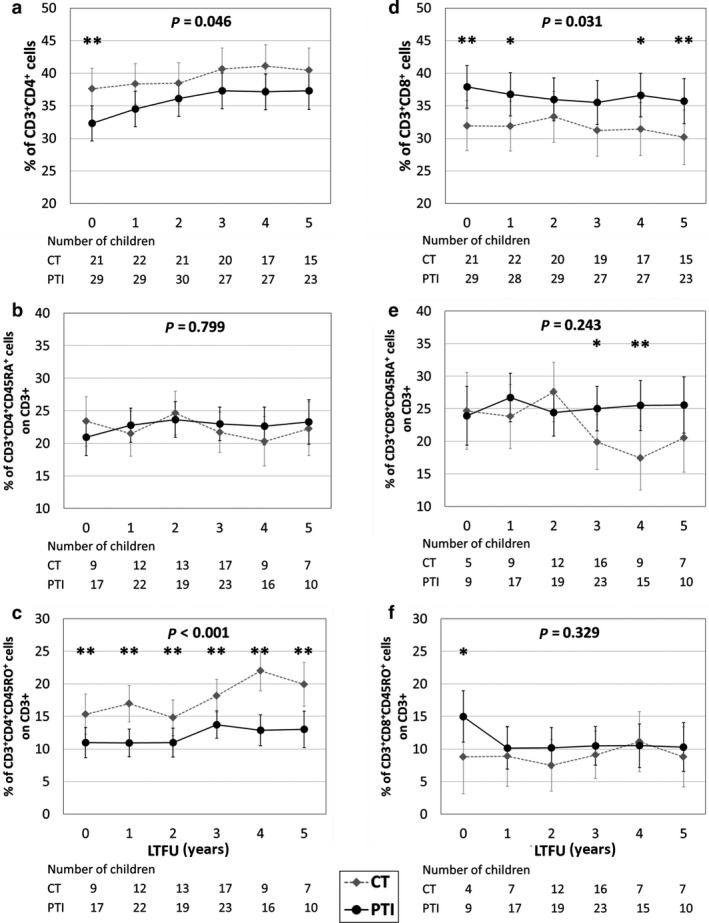
Trends in percentage of CD4, CD8 and their respective memory and naïve subpopulations during 5 years of long‐term follow‐up (LTFU) in children on continuous antiretroviral (ART) therapy (CT) and those undergoing planned treatment interruption (PTI) of ART. (a–c) Percentage of CD4 (a), CD4‐naïve (b) and CD4 memory (c) cells in CT and PTI children. (d–f) Percentage of CD8 (d), CD8‐naïve (e) and CD8 memory (f) cells in in PTI and CT children. **P < 0.05; *P < 0.10; *P*‐values in panel: overall difference between PTI and CT groups. Whiskers represent 95% confidence intervals of mean values.

The percentage of CD4 cells was significantly lower in PTI than in CT (*P* = 0.012) at the end of the main trial. This difference persisted during long‐term follow‐up (overall, *P* = 0.046), but by 5 years was not statistically significant (Fig. 2a). This reduction in the proportion of CD4 cells was due to a reduction in the CD4 memory cells, which were significantly depleted in the PTI group at the end of the main trial (*P* = 0.027), persisting throughout the study period and at 5 years (overall, *P *˂ 0.001; Fig. 2c). The percentages of CD4‐naïve cells did not differ significantly between PTI and CT (Fig. 2b). At the end of the main trial, the PTI group had a significantly higher percentage of CD8 cells than did the CT group, and percentages of CD8 cells remained higher in PTI than in CT throughout follow‐up (overall, *P* = 0.031). Both naïve and memory CD8 cells contributed to higher CD8% in the PTI children (Fig. 2e‐f).

**Fig. 3:**
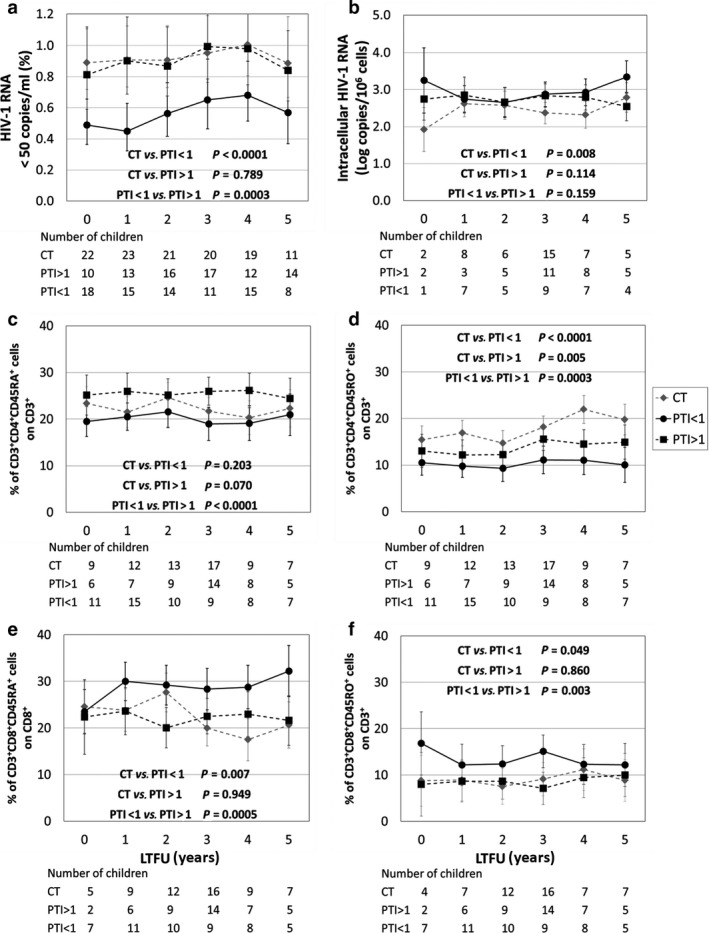
Trends in children on continuous antiretroviral (ART) therapy (CT) and those undergoing planned treatment interruption (PTI) of ART subgrouped by CD4/CD8 ratio during 5 years of long‐term follow‐up (LTFU). (a) Proportion of CT (grey diamond) and PTI children with CD4/CD8 ratio < 1 (PTI < 1), and PTI children with CD4/CD8 ratio >1 (PTI > 1) with undetectable HIV‐1 plasma viraemia. (b) Levels of intracellular HIV‐1 RNA in CT, PTI < 1 and PTI > 1. (c–f) Percentage of CD4‐naïve cells (c), CD4 memory cells (d), CD8‐naïve cells (e) and CD8 memory cells (f) in CT, PTI < 1 and PTI > 1. *P*‐values in panel: paired comparisons between groups.

Mean CD4/CD8 ratio was lower at the end of the main trial in PTI than in CT, and 64% of the PTI arm had CD4/CD8 ratio < 1 compared with 27% of the CT arm; by 5 years corresponding proportions were 36% *vs*. 11%. PTI children with CD4/CD8 ratio < 1 at each time point had a higher proportion of detectable plasma viraemia than did PTI children with CD4/CD8 ratio > 1 and CT children (Fig. 3a); they also had higher levels of intracellular HIV‐1 RNA compared with CT children (Fig. 3b). Children with CD4/CD8 ratio < 1 had significantly lower CD4 memory cells and higher naïve and memory CD8 cells compared with CT children (Fig. 3).

#### Thymic function and immune activation

Levels of TRECs did not differ between PTI and CT children either at the end of the main trial or during follow‐up (Fig. S2a). A trend towards increased expression of CD38 on CD8 cells, a marker of immune activation, was observed in PTI children during therapy interruption [9], which persisted at 1 year but then decreased, with the two groups showing comparable levels during follow‐up (Fig. S2b).

## Discussion

The PENTA 11 trial was undertaken in an era when treatment interruption was being advocated to spare patients from ART, also known as drug conservation [3]. There were also theoretical reasons why cessation of ART might allow host immune mechanisms to ‘control’ HIV‐1 infection [21]. Interruption strategies were then shown to be detrimental in adults and are therefore not recommended in virologically controlled patients. PENTA 11 investigated the response to 48 weeks of CD4‐guided PTI in HIV‐1‐infected children between 2004 and 2008 [8]. The main trial showed that in children interrupting therapy, there were no differences in terms of mortality and clinical outcomes compared with those on continuous ART. Most children exhibited a drop in CD4 count within a month of treatment cessation, with some needing to restart ART because of low CD4. However, in most children, CD4 levels were maintained until ART was re‐introduced at 48 weeks. CD4 nadir and age were important predictors of CD4 count following treatment cessation [8].

In this long‐term follow‐up study, we show that at 5 years after the end of the trial, there were few clinical, virological or immunological differences between the two arms. Both arms had similar rates of undetectable plasma viraemia, HIV‐1 DNA, cell‐associated HIV‐1 RNA and CD4 cell count. One consistent and persistent consequence of PTI was an increase in CD8 cells, which occurred within 8 weeks of ART cessation [8]; CD8 cells remained elevated during ART interruption and, although decreasing again following treatment re‐initiation, were still elevated at 5 years, with the model predicting that they would remain elevated for many years and indeed may never return to the levels in the CT group. Increased CD8 count was an important driver of reduced CD4/CD8 in the PTI arm even after reintroduction of ART. In adults with low CD4/CD8 ratio, the CD8 memory compartment is particularly expanded [19]. In our children with low CD4/CD8 ratio both naïve and memory CD8 cells were expanded. Residual viral replication, outlined by the detection of intracellular HIV‐1 RNA, may drive reduced CD4/CD8 ratio, with particular depletion of CD4 memory cells, target of HIV infection, as well as expansion of CD8 cells.

Our models suggest that by around 7 years, CD4/CD8 should be similar between the two arms, indicating that PTI does not appear to irreversibly influence the long‐term CD4/CD8 ratio. Significant predictors of CD4/CD8 recovery in the PTI arm included trial baseline CD4/CD8 ratio, time since ART re‐initiation and minimum CD4/CD8 ratio during PTI.

We previously reported that the initial fall in CD4 cell count in the first weeks off ART was due to decrease in both naïve and memory cells with accompanying increase in CD8 memory cells. CD4 count was subsequently maintained in most patients [9]. An interesting observation seen in this sub‐study with longer follow‐up was persisting lower CD4 memory cells in the PTI arm. This was despite maintenance of thymic output, as indicated by naïve CD4 cell recovery and TRECs. The approach used to identify naïve and memory T cells was sustained throughout the entire study. Whilst CD45RO memory cells can re‐express CD45RA, and therefore potentially overestimate naïve T‐cell subsets and underestimate memory cells, we found very low levels of this population, supporting our findings. Residual viral replication may only partially explain this observation. It may indicate a persistent redistribution of CD4 memory cells from circulation into lymphoid tissue. It may also indicate an ongoing requirement for maintaining memory cell involvement in controlling chronic infections, including HIV‐1. Studies are required to observe future changes in memory cell dynamics as well as to fully understand the causes and consequences in children who have experienced treatment interruptions.

Although limited by small sample size and non‐random selection of sub‐study participants, overall our study shows that after PTI children had limited long‐term clinical, virological or immunological consequences. This contrasts with most studies performed in adults [22]. The reasons for this observation remain to be fully determined but may be driven by the extensive capacity for children to maintain and indeed improve immune function through high rates of thymic output.

## Conclusions

In conclusion, long‐term follow‐up data indicate that baseline factors, particularly levels of CD4 and CD8 cells, may be the most important determinants of response to treatment interruption. Results of this study are important, considering that, although PTI alone cannot be recommended in current patient management, it may be included in future therapeutic strategies utilizing novel therapeutic agents, such as therapeutic vaccines and broadly neutralizing antibodies.

## Author contributions

AC, YS, DG, ADR and CG contributed to study conception and design. TRC, AC, YS, YR, CG, ADR, RF and MZ contributed to the acquisition of data and management of samples. DF analysed the main trial data. PDB, RF, ADR and AD contributed to the analysis of sub‐study data. LH contributed to study management. AB, DF, NK, DG, ADR, RF and AD were involved in drafting the manuscript. All authors were involved in critical review of the manuscript and approved the final version.

## Supporting information

**Fig. S1.** Trends in CD4 percentage, CD4 cell count, CD8 percentage, CD8 cell count, CD4/CD8 ratio and HIV RNA from end of main trial.
**Fig. S1.** Trends of TREC and immune activation levels during five years of long‐term follow‐up in CT and PTI.

**Table S1.** Characteristics at baseline for main study.
**Table S2.** Characteristics at baseline by inclusion in sub‐study.
