# Supplementary material for: Long‐term clinical, virological and immunological outcomes following planned treatment interruption in HIV‐infected children
Source: HIV Med. 2020 Oct 29;22(3):172–84. doi: 10.1111/hiv.12986 (PMC8436743; doi:10.1111/hiv.12986)

**Supplementary Figure 1**: Trends in CD4 percentage, CD4 cell count, CD8 percentage, CD8 cell count, CD4/CD8 ratio and HIV RNA from end of main trial. Trends in CD4 percentage, CD4 cell count, CD8 percentage, CD8 cell count and CD4/CD8 ratio are adjusted for baseline measurements; estimated mean values are presented corresponding to the overall respective mean baseline value for CT and PTI arms combined.


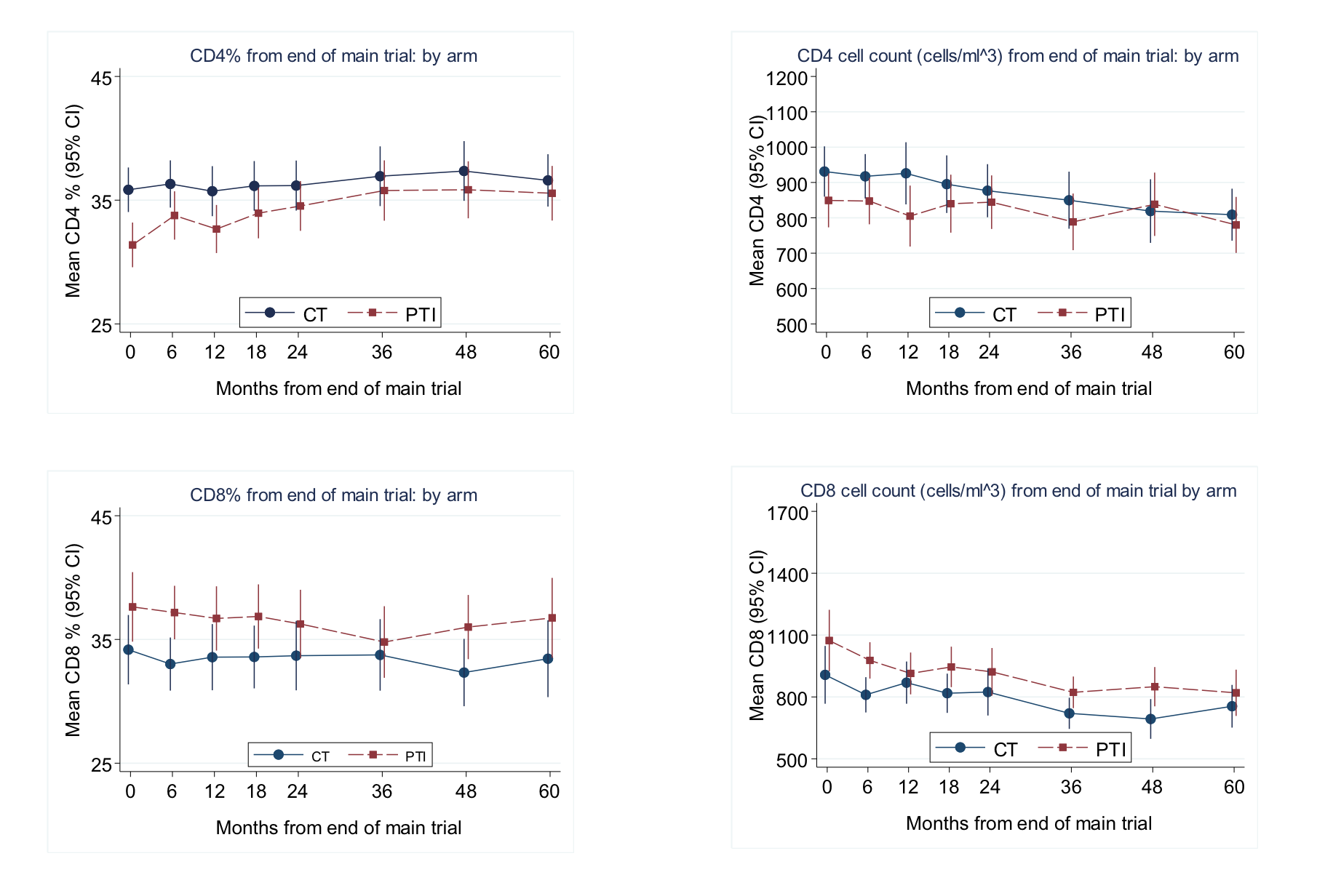


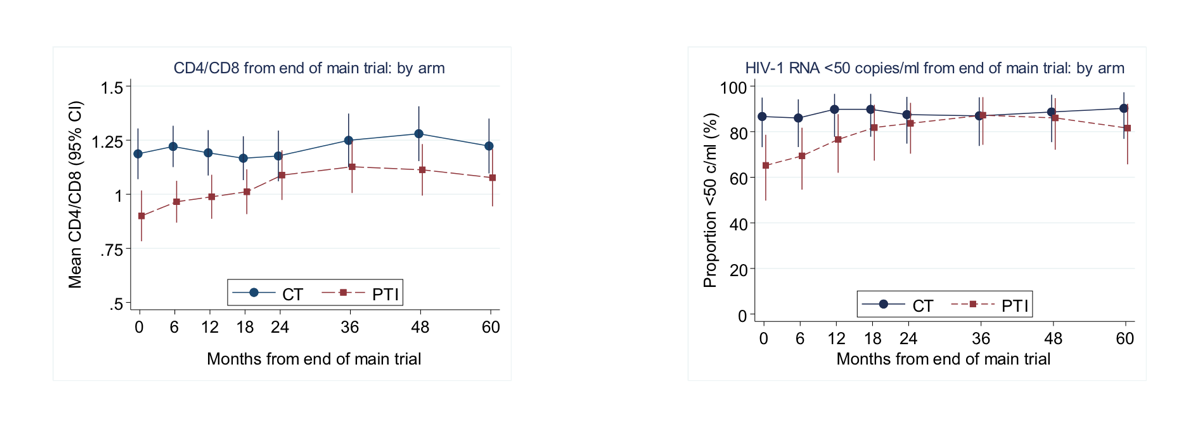


**Supplementary Figure 2.** Trends of TREC and immune activation levels during during five years of long-term follow-up in CT (grey diamond) and PTI (black circle) children. A) TREC copies. B) Percentage of activated CD8CD38 cells. *p<0.10; p values in panel: overall difference between PTI and CT groups. Whiskers represent 95% confidence interval of mean values.


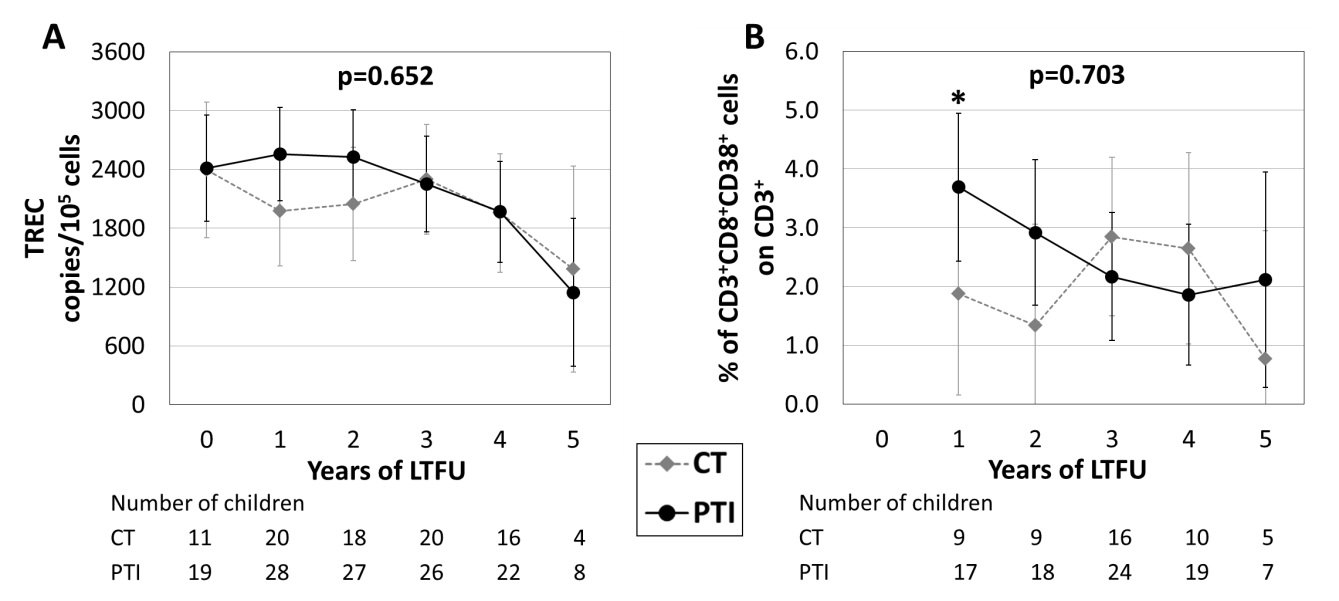

Supplement: Supplementary file 1 — Fig. S1. Trends in CD4 percentage, CD4 cell count, CD8 percentage, CD8 cell count, CD4/CD8 ratio and HIV RNA from end of main trial. Fig. S1. Trends of TREC and immune activation levels during five years of long‐term follow‐up in CT and PTI. [file HIV-22-172-s001.docx]
