# Supplementary material for: Long‐term clinical, virological and immunological outcomes following planned treatment interruption in HIV‐infected children
Source: HIV Med. 2020 Oct 29;22(3):172–84. doi: 10.1111/hiv.12986 (PMC8436743; doi:10.1111/hiv.12986)
Supplement: Supplementary file 2 — Table S1. Characteristics at baseline for main study. Table S2. Characteristics at baseline by inclusion in sub‐study. [file HIV-22-172-s002.docx]

**Supplementary Table 1 Characteristics at baseline for main study**

|  | **At baseline** | |
| --- | --- | --- |
|  | **CT (n=51)** | **PTI (n=50)** |
| Male (%) | 21 (41%) | 24 (48%) |
| Age (years), median (IQR) | 9.8 (6.3, 12.0) | 8.8 (6.1, 11.4) |
| Ethnic origin (%) |  |  |
| White | 16 (31%) | 20 (40%) |
| Black | 16 (31%) | 14 (28%) |
| Asian | 12 (24%) | 10 (20%) |
| Other | 7 (14%) | 6 (12%) |
| CDC disease stage |  |  |
| N | 8 (16%) | 9 (18%) |
| A | 11 (22%) | 16 (32%) |
| B | 13 (25%) | 16 (32%) |
| C | 19 (37%) | 9 (18%) |
| Baseline weight for age (z score), median (IQR) | 0.2 (-1.4, 0.9) | -0.5 (-1.1, 0.2) |
| Age when started ART (years) | 2.2 (0.4, 4.6) | 1.8 (0.5, 4.3) |
| Cumulative ART exposure (years), median (IQR) | 6.0 (3.9, 8.8) | 5.6 (3.2, 8.3) |
| CD4 parameters, median (IQR) |  |  |
| CD4 % of total lymphocytes | 37 (34, 40) | 37 (33, 43) |
| CD4 (cells/mm^3^) | 965 (738, 1222) | 1013 (860, 1280) |
| Nadir CD4% before starting ART | 18 (10, 27) | 21 (12, 26) |
| CD8 parameters, median (IQR) |  |  |
| CD8 % of total lymphocytes | 30 (26, 34) | 32 (24, 37) |
| CD8 (cells/mm^3^) | 790 (576, 1020) | 851 (609, 1072) |
| CD4/CD8 ratio, median (IQR) | 1.24 (1.00, 1.48) | 1.17 (0.97, 1.45) |
| CD4/CD8 ratio less than 1 | 12 (24%) | 14 (28%) |
| HIV RNA<50 copies/mL | 48 (94%) | 43 (86%) |

**Supplementary Table 2: Characteristics at baseline by inclusion in sub study**

|  | **CT arm** | | | **PTI arm** | | |
| --- | --- | --- | --- | --- | --- | --- |
|  | **Not in sub study** | **In sub study** | **P*** | **Not in sub study** | **In sub study** | **P*** |
|  | **N=28** | **N=23** |  | **N=19** | **N=31** |  |
| Male (%) | 12 (43%) | 9 (39%) | 1.0 | 10 (53%) | 14 (45%) | 0.78 |
| Age (years), median (IQR) | 8.6 (6.0, 12.7) | 10.1 (7.0, 11.1) | 0.65 | 8.2 (6.7, 11.4) | 9.0 (6.0, 10.9) | 0.87 |
| Ethnic origin (%) |  |  |  |  |  |  |
| White | 7 (25%) | 9 (39%) |  | 6 (32%) | 14 (45%) |  |
| Black | 8 (29%) | 8 (35%) |  | 2 (11%) | 12 (39%) |  |
| Asian | 12 (43%) | 0 (0%) |  | 10 (53%) | 0 (0%) |  |
| Other | 1 (4%) | 6 (26%) | <0.001 | 1 (5%) | 5 (16%) | <0.001 |
| CDC disease stage |  |  |  |  |  |  |
| N | 4 (14%) | 4 (17%) |  | 5 (26%) | 4 (13%) |  |
| A | 5 (18%) | 6 (26%) |  | 5 (26%) | 11 (35%) |  |
| B | 8 (29%) | 5 (22%) |  | 5 (26%) | 11 (35%) |  |
| C | 11 (39%) | 8 (35%) | 0.89 | 4 (21%) | 5 (16%) | 0.59 |
| Baseline weight for age (z score), median (IQR) | -0.3 (-1.6, 0.7) | 0.2 (-1.4, 1.2) | 0.23 | -0.7 (-1.7, -0.5) | -0.2 (-0.8, 0.3) | 0.02 |
| Age when started ART (years) | 2.5 (1.0, 7.0) | 0.6 (0.3, 4.3) | 0.08 | 2.4 (0.9, 6.7) | 1.3 (0.4, 3.3) | 0.06 |
| Cumulative ART exposure (years), median (IQR) | 4.1 (2.9, 7.9) | 7.1 (5.3, 9.0) | 0.01 | 3.2 (2.7, 7.0) | 6.3 (4.0, 8.3) | 0.09 |
| CD4 parameters, median (IQR) |  |  |  |  |  |  |
| CD4 % of total lymphocytes | 36 (32, 38) | 40 (35, 43) | 0.02 | 36 (32, 40) | 38 (33, 42) | 0.39 |
| CD4 (cells/mm^3^) | 910 (688, 1068) | 1060 (710, 1230) | 0.48 | 1028 (829, 1310) | 917 (839, 1086) | 0.37 |
| Nadir CD4% before starting ART | 16 (8, 20) | 24 (16, 28) | 0.03 | 19 (13, 30) | 21 (10, 25) | 0.97 |
| CD8 parameters, median (IQR) |  |  |  |  |  |  |
| CD8 % of total lymphocytes | 31 (27, 34) | 30 (25, 36) | 0.79 | 33 (28, 41) | 31 (24, 35) | 0.14 |
| CD8 (cells/mm^3^) | 772 (566, 1047) | 790 (576, 940) | 0.73 | 945 (760, 1386) | 685 (517, 1027) | 0.06 |
| CD4/CD8 ratio, median (IQR) | 1.11 (0.93, 1.44) | 1.27 (1.08, 1.56) | 0.20 | 1.03 (0.85, 1.33) | 1.27 (1.05, 1.51) | 0.05 |
| CD4/CD8 ratio below 1 (%) | 9 (32%) | 3 (13%) | 0.18 | 8 (42%) | 6 (19%) | 0.11 |
| HIV RNA<50 copies/mL | 27 (96%) | 21 (91%) | 0.58 | 18 (95%) | 25 (81%) | 0.23 |

*Test for difference: included in sub study vs. not included in sub study (Fisher’s exact test or Ranksum test)
